# Redefining Meaning: A Micro-Genetic Model of the Constitution of Experience

**DOI:** 10.1007/s12124-024-09849-5

**Published:** 2024-05-22

**Authors:** Matteo Reho, Sergio Salvatore

**Affiliations:** 1 Department of Dynamic and Clinical Psychology, and Health Studies, Sapienza University of Rome, Rome, Italy; 2 Department of Human and Social Sciences, University of Salento, Lecce, Italy

**Keywords:** Meaning-making, Value of life, Sign, Pertinentization, Significance in praesentia, Significance in absentia

## Abstract

This article aims to renew the discussion about meaning in the field of psychology. A model is presented that, contrary to the classical view of meaning as an entity taken for granted, explains the dynamics through which it comes to be constituted, opening itself to the possibility of being experienced, as a psychological reality. The autoethnographic analysis carried out by von Fircks (IBPS 53(4):632–643, 2023) is used as example to show how such a model enables an understanding of local phenomena through the comprehension of the semiotic dynamics underlying them. Finally, this paper offers insights into the mechanisms that underlie the field of possibility of meaning-making processes, thus of human experience.

## Introduction

Contemporary psychology implicitly assumes a view of meaning as a already given entity, standing before the social dynamics associated with it (Salvatore, 2016). Such a view of meaning implies a renounce of the understanding of how meaning emerges, in so doing limiting the field of psychological inquiry. In the final analysis, contemporary psychology’s focus on how signs, as vehicles of meaning, are processed/manipulated by the mind implies the impossibility of understanding the genesis of meaning as a psychological reality (Salvatore, 2012).

Bruner (1990) must be recognized for bringing the question of meaning back within psychological discourse. For the American psychologist, cognitive processes have not to be understood as mechanisms endowed with internal rules, but rather as the ways through which people act out their being in the world; that is, they signify experience (Bruner, 1990). Such a view reverses cognitivism’s logic: mental processes do not function as mere elaboration of information already present in the external environment; rather, they are the way through which the environment is made meaningful (Salvatore, 2019; Valsiner, 2014).

This paper intends to contribute to fostering a reprise of the discussion of the genesis of meaning by recalling some fundamental issues of the constitution of experience (De Luca Picione & Salvatore, 2023; Salvatore et al., 2022). Such a model represents, on the one hand, a distance from the traditional conception of meaning assumed in the field of psychology; on the other hand, it offers insights for comprehending the mechanisms underlying the field of possibility of meaning-making processes.

### The Centrality of Meaning in the Constitution of Experience

The relevance of meaning in human life has been recognized by a number of disciplines such as sociology (e.g., Berger & Luckmann, 1966), linguistics (e.g., Linell, 2009), anthropology (e.g., Douglas, 1986), to name a few. The conception of meaning assumed in psychology has its roots in the dyadic theory of de Saussure (1916), who conceived of meaning as the idea contained in and conveyed by the sign, that is, the union of signifier (content) and signified (expression) (Eco, 1975). This view conceives meaning as an autonomous entity; as if, for example, the meaning of a gesture or word were contained in the gesture or word itself. Experience, then, would be a mirror of meanings which pre-exist in the reality as given entities. In contrast, Bruner (1990) recovered a view according to which experience would not reproduce the objects of reality as if they were given entities but would be established by means of meaning, which would operate as a filter, foregrounding some components of reality and backgrounding the others (Bickhard, 2009; Manzotti, 2010). This view has led to the development of models of the mind based on meaning (e.g., Salvatore, 2016, 2018; Valsiner, 2007, 2014). According to these models, the meaning is to be understood as the mind’s peculiar way of making some elements of reality relevant, of giving them a form, thus bringing them out as contents of experience (Salvatore et al., 2022).

Such a view of meaning as constitutive of experience is not new; in fact, it is based on a long philosophical and psychological tradition (e.g., Kant, Husserl, Gestalt theory) that understood the contents of experience (e.g., gestures, words, events, feelings, etc.) as emerging from basic mental processes that organize sensory input into meaningful forms for the subject, rather than as primitive elements (De Luca Picione & Salvatore, 2023). According to the perspective adopted in this paper, the characteristic feature of the mind would be precisely the foregrounding-backgrounding process that would reflect human variability. In brief, seeing the piece of paper or the banknote, the glass half-full or half-empty, the forest or the trees, would reflect the subjective act of shaping the content of the experience.

### The Value of Life of Meaning

According to the perspective proposed, the internal representations emerging from the foregrounding-backgrounding process must be understood as having subjective cogency: namely, *value of life* (Salvatore, 2012).

Meaning-making concerns signs (i.e., something that stands for something else); however, people experience them as concrete entities in the world, rather than as something that stands for it (see next paragraph). In other words, meaning-makers treat the sign as if it were the thing it represents and not as something that represents something else which is absent (Salvatore, 2019). According to this perspective, then, the mind-world relationship is always indirect; it occurs through the mediation of signs that enable people to relate to the object of experience. However, the value of life of signs (and thus of meaning) emerges when – and in terms of – the mind-world relation is perceived as direct; that is, it is treated as if it were not mediated by signs, as if experience emerged from the direct relation with the world (Salvatore, 2016).

Of note, the disappearance in the subject of the sign-mediated action – from which the value of life emerges – is not a constant condition. Indeed, many circumstances involving existentially relevant objects of experience can be experienced without value of life; at the same time, some circumstances that have no reference to facts and states of reality can on the contrary be experienced as having value of life (Salvatore, 2012). Consider, for example, how war-related events (e.g., bombings, migratory flows, genocides) are perceived by some people as purely abstract concepts, something void of existential power, therefore to which they remain indifferent (i.e., lacking value of life). Conversely, think of something not endowed with substantial consistency such as a country. If a person forgets to apply for a visa, he or she will not be able to enter a country not because it will be the latter that prevents him or her from doing so, but rather because of a border police officer who performs his or her function by assuming the country as an existential entity (i.e., endowed with value of life). These examples show, as argued by Meinong (Albertazzi & Jacquette, 2001), the independence of the psychological object from its ontological status. Once this is recognized, it is central to understand *how* the mind acts to provide psychological reality (i.e., value of life) to its contents.

### A Micro-Genetic Model of Meaning

According to Peirce’s (1897/1932) triadic theory, meaning is the effect of sign. Contrary to the dyadic theory proposed by de Saussure, Peirce argues that the meaning of a sign is not conveyed by the sign itself; rather, it acquires its meaning according to the subsequent sign. For example, it is because of sign B, activated by sign A in the person’s mind, that sign A acquires meaning. The interpretation that sign B elicits of sign A consists in defining the *“respect or capacity”* (Peirce, 1897/1932, p. 228) of the object that sign A represents (Peirce defines *ground* such “respect or capacity”). Meaning is thus determined by signification (i.e., by the sign A-sign B relationship) and is therefore something abstract; what actually exists is the continuous flow of signs (Salvatore, 2016). Moreover, meaning is contextual in that it emerges from the network of relationships between signs that precede and follow in the contingency of the present moment (Salvatore, 2013). Finally, the meaning is bivalent. The interpretation of the preceding sign by the following one has to do with what Peirce called ground (i.e., the aspect or capacity), that is, the quality for which the preceding sign stands for the object (Peirce, 1897/1932). It follows that the extracted ground represents the meaningful aspect of the object to be represented at the expense of the infinite possible grounds in terms of which the object could be signified (Salvatore et al., 2022). For example, stating that Matthew is tall implies, on the one hand, establishing as ground the quality of height; on the other hand, putting in the background all the other possible grounds (e.g., profession [the fact that Matthew is a student], gender [the fact that Matthew is a man], age [the fact that Matthew is young], etc.). Salvatore (2016) called *Significance in Praesentia* (SIP) and *Significance in Absentia* (SIA) the dual valence of the sign, where the SIA, rather than being what is said about the object, is the set of infinite potential grounds that what is said backgrounds. Accordingly, it is in the dialectical relationship between SIP and SIA that the content of the experience is constituted and the meaning-maker is enabled to think of it.

At the representational level, the signification (i.e., the way by which meaning is determined) can be distinguished into dynamics, structure and content (Salvatore & Cordella, 2024). Dynamics concerns how signs combine with each other over time and doing so they let the meaning emerge. Structure represents the organization of dynamics; that is, the constraints that channel the selection of signs that enter the semiotic chain (i.e., the ground). The ground therefore lends itself to being represented in terms of its decomposition into elementary components, each of which describes a part of the variability with which signs combine with each other (Salvatore & Venuleo, 2013). It is worth adding that structure and dynamics are not separate, but rather circular; that is, structure organizes dynamics and the latter in its unfolding reproduces structure (Salvatore & Cordella, 2024).

### The Autoethnographic Analysis and the Micro-Genesis of its Conditions of Exercise

To clarify the above, let us take the work published by von Fircks (2023) as example. The author highlights the tendency of mindfulness research to neglect the theoretical and historical foundations that inform it. Von Fircks attempts to fill this gap by proposing the integration of the philosophical principles of Taoism with Mead’s theory as the conceptual framework to model mindfulness. The author uses an autoethnographic analysis to demonstrate that through meditation the individual can experience the emergence of a new I able to relate to the social environment differently from the old I. Von Fircks reads the change that occurred in his stream of thoughts in light of the integrated theory he proposed and states that, through the meditative act, he became aware of an achievement-oriented I, overly focused on results and success defined by exogenous standards. Moreover, he realized that this focus did not allow him to achieve inner equilibrium as it limited him to focus on one pole of what he calls the “Taoist equation” (progress and acceleration), leaving the opposite pole (de-acceleration and temporary decline) in the background. Meditation, therefore, enabled him to integrate the hitherto unconsidered polarity and endorse a new I, centered on inner needs, rather than exogenous achievements.

**Fig. 1:**
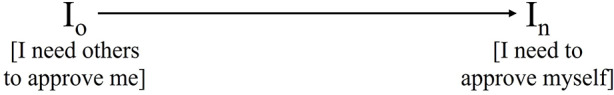
The change in the content of the experience enabled by the meditation. *Note* I_o_= old-I; I_n_= new-I

Von Fircks’ autoethnographic analysis can help to epitomize the distinction between Significance in Praesentia and Significance in Absentia and the hidden semiotic dynamics that must be considered for a deeper understanding of the subjective experience. From the standpoint of the author/subject of the exercise of meditation, he was able to recognize a certain form of itself (the old-I) and to move toward a different form (the new-I), channeled in that by the trajectory of sense provided by the Taoist equation (i.e., the juxtaposition between the progress-acceleration and the de-acceleration-temporary decline polarities). Figure 1 detects this process of change in the content of the experience: the self-representation as someone seeking approval by others as a result of the capacity of accomplishment of socially defined standards (the old-I) to the self-representation as someone focused on his own approval as a result of the capacity to fulfill his own inner needs.

Now, such a change in the content of the experience detected by the autoethnographic description tells a relevant part of the story – the one substantiating the subject’s feelings and beliefs. Yet, this is only a part – namely, the part that speaks about what happens once the contents of the experience have emerged and thus lend themselves to be elaborated by the subject. In other terms, the old-I from which the change in the new-I starts is not the actual starting point of the whole meaning-making process. Indeed, the old-I is the output of a hidden micro-genetic semiotic process (the Significance in Absentia) that makes such a representation be constituted as a content experienced as a mental object.

**Fig. 2:**
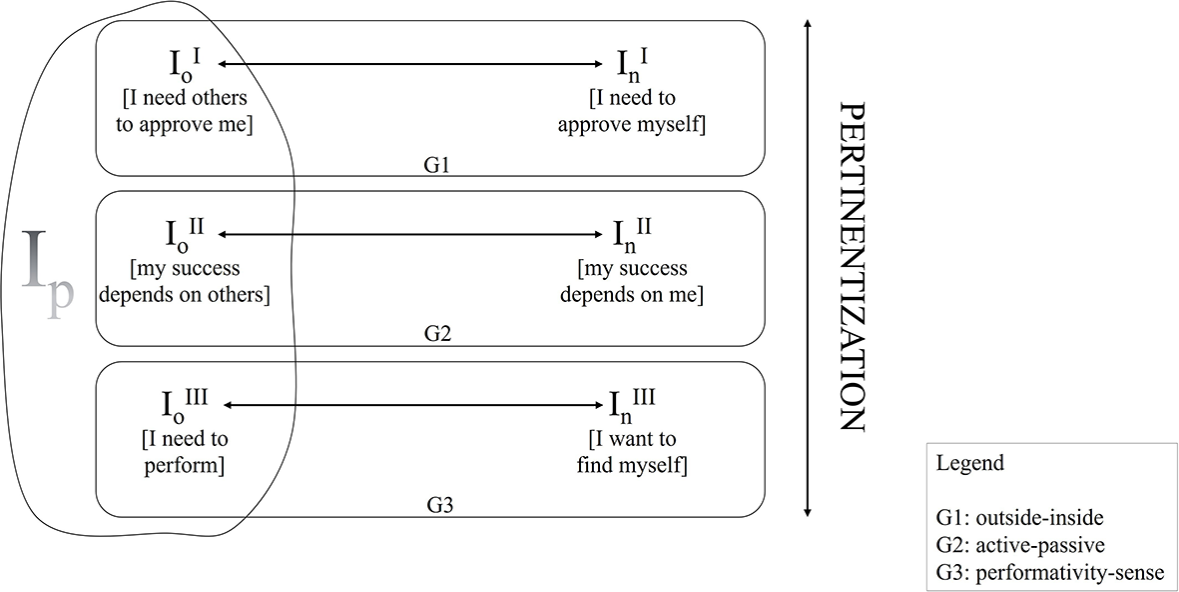
The micro-genetic emergence of the content of the experience which meditation acts on. *Note* I_o_= old-I; I_n_= new-I. The Roman numerals represent three potential old-I/new-I dialectics that may emerge according to the ground pertinentized

Figure 2 illustrates such a micro-genetic semiotic process. In the beginning, there is not an already defined representation. Rather, there is an infinite set of potential representations. To limit to the case discussed here, the author’s self-representation, one must assume that the self is an infinite set of possible representations (I_p_) (a field-sign, according to Valsiner [2007] terminology). Each of these representations consists of a specific ground (i.e., a given “respect or capacity”) put to the fore (i.e., made pertinent; on the role of the pertinentization in the micro-genetic emergence of meaning, see Salvatore et al., 2022). Thus, the content of the experience is made up by such a process of pertinentization of the ground. Accordingly, one can imagine as many representations of the self – i.e., many “I” – as the potentially infinite grounds are. Figure 2 exemplifies three bipolar grounds – the one from which the old-I—new-I described by the autoethnographic analysis (outside vs. inside) emerges and two further possible grounds: |active vs. passive| and |performativity vs. sense|. According to the pertinentized ground, it can be the case that the outside-centered old-I emerges [I need others to approve me] as the starting point of the change in the inside-centered new-I [I need to approve myself], as well as the performance-centered old-I [I need to perform] open to change in the sense-oriented new-I [I want to find myself].

What is relevant to note is that the author’s very possibility of self-representing himself in terms of an achievement-oriented old-I to change in the inner needs-centered new-I is already the output of a micro-genetic semiotic dynamics of pertinentization of one of the infinite possible grounds. The ground made pertinent works as the Significance in Absentia that, latently, makes a radical reduction of the infinite potentiality of what can be represented. As a result, a component of such a potentiality is constituted as the content of the experience, ready to be elaborated – in this case, by the meditation practice. And this is the same as saying that, alike the moon, meaning-making has a dark side – the mechanism of the pertinentization that operates from the outside of the realm of the meaning as the genetic dynamics constituting the latter. Accordingly, the comprehension of any meaning-making process must integrate the understanding of the Significance in Praesentia and its elaboration (i.e., in the case discussed, the elaboration consisting of the negative connotation of the search for achievement and the related investment in changing such a search in the juxtaposed search for satisfaction of inner needs) with the understanding of the Significance in Absentia, namely the dynamics of pertinentization of the ground the meaning emerges from (for a model of such a dynamics, see Salvatore et al., 2022).

Thus, to sum up, meditation – more generally, any form of mental act of feeling and thinking – can operate because of and within the constraints of the dynamics of pertinentization of the ground in which the subject is embedded and by which, in the final analysis, it is constituted.

## Conclusion

The purpose of this paper was to emphasize the need to enlarge the view of meaning as a taken-for-granted entity to encompass the fundamental genetic dynamic that leads meaning to be constituted, and experienced, as a psychological reality. In this regard, some fundamental issues underlying a micro-genetic model of the constitution of meaning – thus of experience – were recalled, providing some insights for the understanding of the mechanisms underlying the field of possibility of meaning-making processes. Finally, this paper is intended to act as an impulse for a renewed discussion about the concept of meaning and its central role in the study of psychological life.

## Data Availability

No datasets were generated or analysed during the current study.
